# Computational Prediction and Validation of BAHD1 as a Novel Molecule for Ulcerative Colitis

**DOI:** 10.1038/srep12227

**Published:** 2015-07-17

**Authors:** Huatuo Zhu, Xingyong Wan, Jing Li, Lu Han, Xiaochen Bo, Wenguo Chen, Chao Lu, Zhe Shen, Chenfu Xu, Lihua Chen, Chaohui Yu, Guoqiang Xu

**Affiliations:** 1 Department of Gastroenterology, the First Affiliated Hospital, College of Medicine, Zhejiang University, Hangzhou, China; 2 Department of Gastroenterology, Peking University People’s Hospital, Beijing, China; 3 Beijing Institute of Radiation Medicine, Beijing, China

## Abstract

Ulcerative colitis (UC) is a common inflammatory bowel disease (IBD) producing intestinal inflammation and tissue damage. The precise aetiology of UC remains unknown. In this study, we applied a rank-based expression profile comparative algorithm, gene set enrichment analysis (GSEA), to evaluate the expression profiles of UC patients and small interfering RNA (siRNA)-perturbed cells to predict proteins that might be essential in UC from publicly available expression profiles. We used quantitative PCR (qPCR) to characterize the expression levels of those genes predicted to be the most important for UC in dextran sodium sulphate (DSS)-induced colitic mice. We found that bromo-adjacent homology domain (BAHD1), a novel heterochromatinization factor in vertebrates, was the most downregulated gene. We further validated a potential role of BAHD1 as a regulatory factor for inflammation through the TNF signalling pathway *in vitro*. Our findings indicate that computational approaches leveraging public gene expression data can be used to infer potential genes or proteins for diseases, and BAHD1 might act as an indispensable factor in regulating the cellular inflammatory response in UC.

Ulcerative colitis (UC) and Crohn’s disease (CD), the two main subtypes of inflammatory bowel disease (IBD), are immunologically mediated, idiopathic, chronic and relapsing diseases^1^, the aetiology of which remains unclear. From the latest Asian epidemiological investigation, the incidence of IBD, especially UC, is rising in parallel with the rapid socioeconomic development and westernization of lifestyle during the past two decades^2^,^3^.

Intestine epithelial cells (IECs), a layer of which forms a physical barrier separating subepithelial mucosal immune cells from a variety of antigenic substances present in the intestinal lumen, play a pivotal role in maintaining a balanced intestinal microenvironment^4^. Disruption or dysfunction of the intestinal barrier promotes contact between immune cells and antigens, and the excessive cytokines produced are involved in the pathogenesis of UC, causing mucosal tissue destruction and leading to bloody diarrhoea^5^. The inflammatory responses that mediate inflammatory signalling are driven by classical stimulus-regulated transcription factors, including nuclear factor κ B (NF-κ B), activator protein-1(AP-1), IFN regulatory factors (IRFs)^6^ and mitogen-activated protein kinases (MAPKs)^7^.

Gene expression microarrays, which are frequently and widely applied in clinical studies of human diseases, enable the measurement of genome-wide expression^8^. This method has been applied to investigate transcriptional signatures present in gastrointestinal tissue obtained from CD and UC patients for more than 10 years^9^. However, the majority of the genes identified by this method do not necessarily play critical roles in the biological processes under investigation. For this reason, we adopted a systematic computational in silico approach to predict novel genes or proteins on the basis of comprehensive testing of molecular signatures in siRNA-disease pairs: a pattern-matching strategy based on GSEA to more precisely select genes to study. GSEA is an analytical method that uses gene sets representing different biological processes to interpret gene expression data, producing a score that measures the similarity between two different processes by comparing their expression profiles^10^. This method has been used in drug discovery with gene sets responsible for diseases to interpret the expression data from different drugs^11^,^12^.

In this study, we estimated the similarity between public expression data derived from UC patients and that from siRNA perturbed cells by applying the parameter ‘insensitive’ and the GSEA systematic algorithm^13^ to estimate the distance between UC and different siRNA perturbations. We also verified the predicted gene expression changes in a mouse model of acute DSS-induced colitis, a canonical IBD model. Our results revealed that the protein BAHD1 is downregulated the most in the colon tissue of the mouse model. As it has not been previously described to have efficacy for UC or any related disorder of inflammation in the gastrointestinal tract, we evaluated the efficacy of BAHD1 in UC and explored its effect on the human epithelial colorectal adenocarcinoma cell line Caco-2 exposed to inflammatory mediators and its related molecular mechanism.

## Results

### Computational Prediction and Assessment of a Novel Target in UC: BAHD1

**Figure 1:**
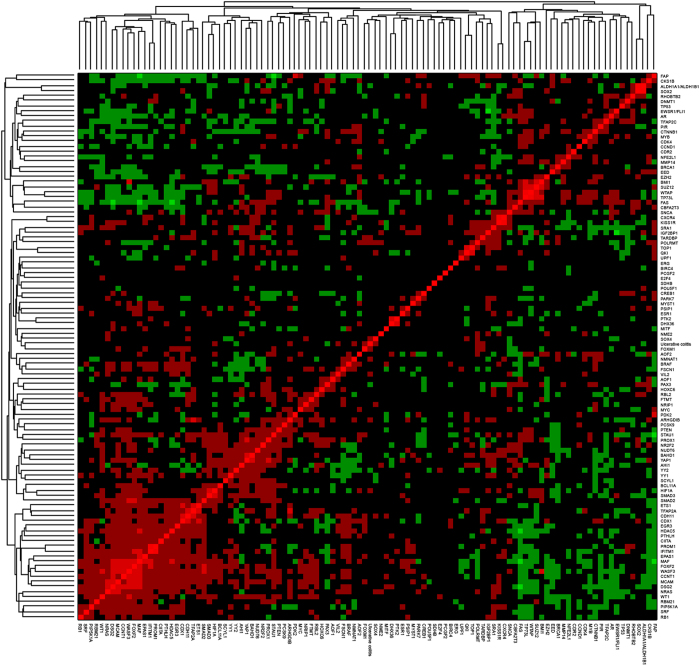
Clustergram of distances between ulcerative colitis and siRNA perturbations.

**Figure 2:**
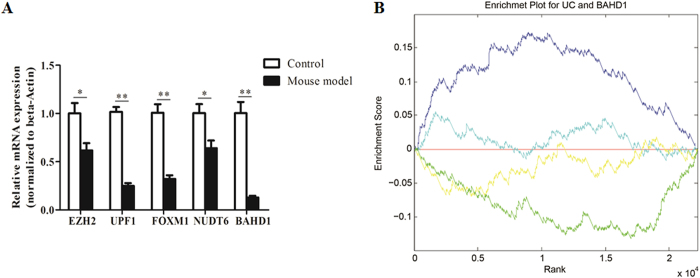
(**A**) qPCR analysis of the five most likely causative genes in the mouse UC model, namely, EZH2, UPF1, FOXM1, NUDT6 and BAHD1. Bars indicate the mean ± SEM; n = 3 per group; *P < 0.05; **P < 0.01. (**B**) Enrichment plot for UC and BAHD1. The enrichment plot presents the hit position information of signature genes in a reference PRL. The size of signature genes is n, and PRL length is N. The function value goes up by 1/n at the hit positions and goes down by 1/(N-n) at the miss hit positions. The line coloured cyan represents the running enrichment score plot of BAHD1 upregulated signature genes in UC’s PRL, and the final enrichment score (i.e., maximum deviation of running enrichment score from zero) is 0.0557(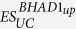). The line coloured yellow represents the running enrichment score plot of BAHD1 downregulated signature genes in UC’s PRL, and the final enrichment score is −0.0706(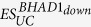). The enrichment score of BAHD1’s signature in UC’s PRL is 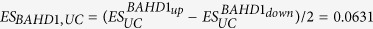. The line coloured blue represents the running enrichment score plot of UC upregulated signature genes in BAHD1’s PRL, and the final enrichment score is 0.1738(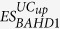). The line coloured green represents the running enrichment score plot of UC downregulated signature genes in BAHD1’s PRL, and the final enrichment score is –0.1331(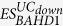). The enrichment score of UC’s signature in BAHD1’s PRL is 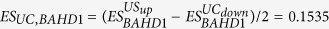. We can infer that the top- (or bottom-) ranked genes in UC are strongly positively (or negatively) regulated in BAHD1, resulting in a high enrichment score for UC’s signature in BAHD1’s PRL.

We calculated the distance values between UC and siRNA perturbation expression profiles based on GSEA. The siRNA perturbation expression profiles represented cell responses to the silencing of as many as 106 different genes (see Methods). By examining the relationships between UC and the 106 siRNAs based on predicted distance scores, we identified major clusters of the entire data set [Fig. 1]. A low distance value indicates a similar gene regulation tendency, and the distance values between host responses to UC and those to siRNA perturbations are shown in Supplementary Table S1. We picked out the top five genes whose single siRNA perturbations had the lowest distance values relative to UC, namely, EZH2, UPF1, FOXM1, NUDT6, BAHD1 (distance value = 0.868, 0.878, 0.883, 0.885, 0.891, respectively), and we investigated the mRNA levels of these most likely candidate genes in the DSS-induced colitis mouse model by qPCR. Among them, BAHD1 was found to be the most downregulated in the mouse model compared with the control group [Fig. 2A], suggesting that this protein might be essential in this inflammatory bowel disease. An enrichment plot showing the enrichment score (ES) for the gene list rank for UC and BAHD1 is shown in Fig. 2B.

### Decreased BAHD1 Expression in *in vivo* and *in vitro* Models and UC Patients

**Figure 3:**
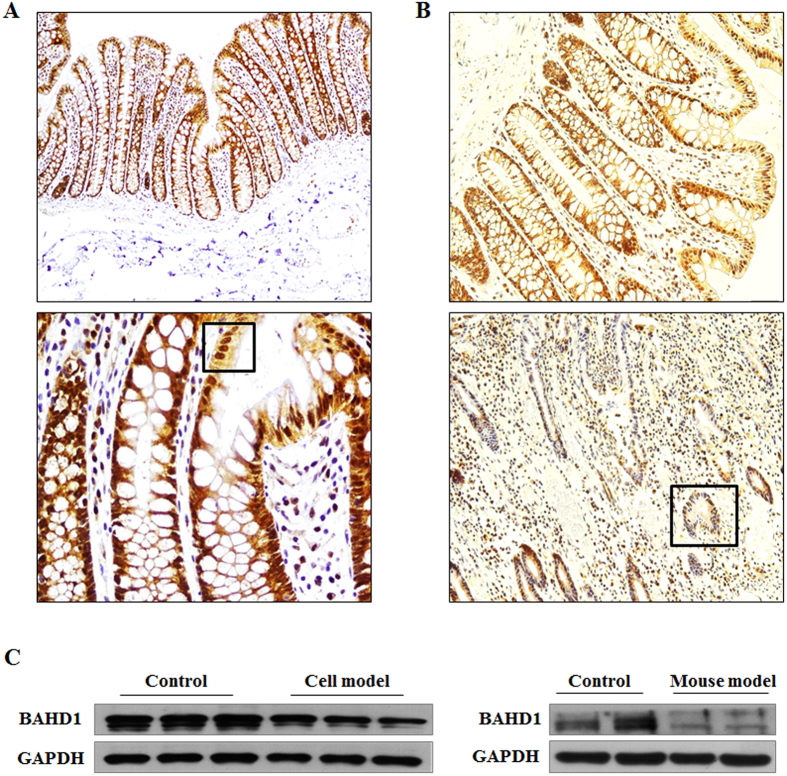
Reduced BAHD1 expression in *in vivo* and *in vitro* models and in UC patients. (**A**) Nuclear localization of BAHD1 in normal human large intestine. Histological sections of colon samples taken from healthy adjacent or distant colon from subjects of certain human cancers were stained for BAHD1. Magnification of 10*10 (upper) and 10*40 (lower) show that BAHD1 (brown staining) is universally present in IECs and crypt cells in the large intestine. (**B**) IHC for BAHD1 in UC patient colonic tissue (lower) compared with the control group (upper), 10*20 magnification. (**C**) Western blot analysis of BAHD1 expression *in vitro* and *in vivo*. The blots shown represent at least three independent experiments; GAPDH was used as a loading control.

To characterize BAHD1 expression in colon tissue, intestinal samples from healthy humans were evaluated by using immunohistochemistry (IHC). As shown in Fig. 3A, BAHD1 protein was expressed normally in the healthy human large intestine, including crypt and epithelial cells, mainly in the nucleus. As for the protein’s expression in colitis, we found that BAHD1 was significantly decreased in IECs and crypt cells in the large intestine of UC patients compared with control patients who had no history of intestinal inflammation [Fig. 3B], indicating that dysregulated expression of BAHD1 in the intestine may be associated with regions of active disease in UC. Western blotting using *in vivo* and vitro models supported this inference: Caco-2 cells showed a significantly reduced level of BAHD1 protein in a cell model, in which Caco-2 cells were exposed to inductive factors for 24 h (see Methods). A similar observation was made in the mouse model of acute colitis (see Methods) in comparison with the control group [Fig. 3C]. Therefore, we next explored BAHD1’s functions and unveiled its possible molecular mechanisms in the cell model, which might give some hints regarding the development of UC.

### Associated Inflammatory Mediators were Enhanced in BAHD1-deficient Caco-2 Cell Model

**Figure 4:**
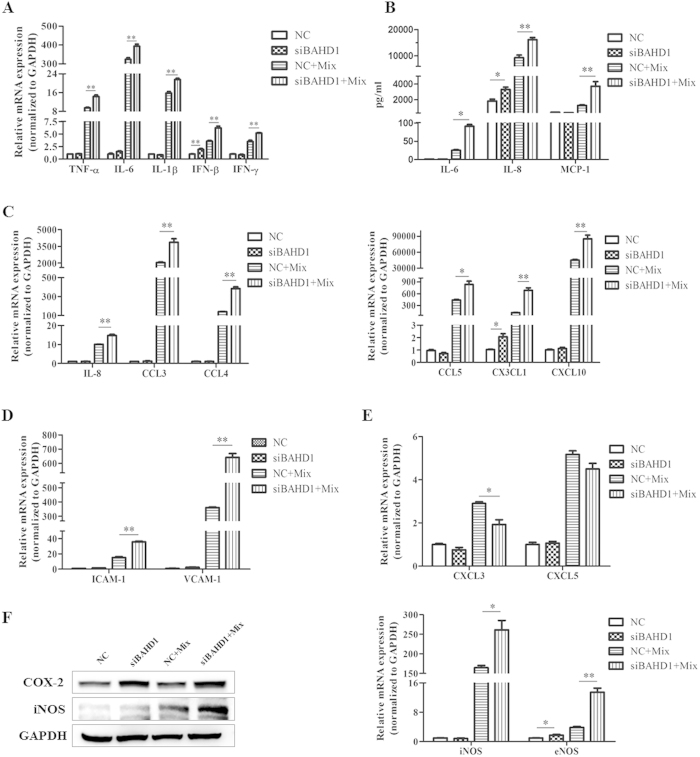
Associated inflammatory mediators were enhanced by BAHD1 deficiency *in vitro*. Four groups are involved here (NC = negative control siRNA-transfected group; NC+Mix = Caco-2 cell model group; siBAHD1 = siBAHD1-transfected group; siBAHD1 + Mix = Caco-2 cell model pre-treated with siBAHD1). (**A**) Effects of BAHD1 repression on the expression of proinflammatory factors (TNF-α, IL-6, IL-1β, IFN-β and IFN-γ) in the Caco-2 cell model. (**B**) Detection of IL-6, IL-8 and MCP-1 secretion in cell culture supernatant by ELISA, the siBAHD1 group is reported as the fold increase compared with the simple cell model. (**C**) BAHD1 inhibition in the cell model influenced the mRNA levels of chemokines such as IL-8, CCL3, CCL4, CCL5, CX3CL1 and CXCL10. (**D**) Expression of the cell adhesion molecules ICAM-1 and VCAM-1 displayed similar increasing trends in the cell model with siBAHD1 interference. (**E**) The expression of CXCL3 and CXCL5 did not show the predicted trend at the mRNA level. (**F**) COX-2 and NO synthases, including iNOS and eNOS, showed higher expression in the cell model pre-treated with siBAHD1. All data above are shown as the mean ± SEM from three independent measurements. Statistical significance was determined by Student’s *t*-test (*P < 0.05; **P < 0.01). The western blots shown are representative of at least three independent experiments.

To explore the relationship between our predicted protein BAHD1 and the responses of IECs in an inflammatory microenvironment, we used the Caco-2 cell line exposed to several inflammatory mediators to establish a cell model to mimic gut inflammation for IECs (see Methods). Caco-2 cells were treated with siBAHD1 for 48 h before 24 h of exposure. The cells were collected for mRNA extraction, and the cell culture supernatant was reserved to measure cytokine secretion. Notably, the cell model pre-treated with siBAHD1 displayed increased mRNA and protein expression of associated cytokines, including pro-inflammatory cytokines such as TNF-α, IL-6, IL-1β, IFN-β and IFN-γ [Fig. 4A,B] and chemokines such as IL-8, CCL3, CCL4, CCL5, CX3CL1 and CXCL10 [Fig. 4B,C]. In addition, certain cell adhesion molecules, including immunoglobulin superfamily intercellular adhesion molecule 1 (ICAM-1) and vascular cell adhesion molecule 1 (VCAM-1), displayed similar increases in the siBAHD1 group [Fig. 4D], and they are important factors mediating leukocyte migration and local inflammation in IBD^14^. However, although most cytokines showed a marked increase in the Caco-2 cell model and even in the siBAHD1 group without any stimulus (like IFN-β and CX3CL1), with decreased expression of BAHD1, several chemokines such as CXCL3 and CXCL5 did not display a similar trend [Fig. 4E]. As for other types of inflammatory mediators, cyclooxygenase-2 (COX-2), an enzyme that catalyses the inflammatory response factor prostaglandin, had higher expression in the cell model pre-treated with siBAHD1, as did two isoforms of nitric oxide (NO) synthases, inducible NOS (iNOS) and endothelial NOS (eNOS) [Fig. 4F].

### Activation of Ikappa B (IκB) Kinase (IKK)/NF-κ B and JNK/AP-1 Pathways in the siBAHD1-treated Caco-2 Cell model

**Figure 5:**
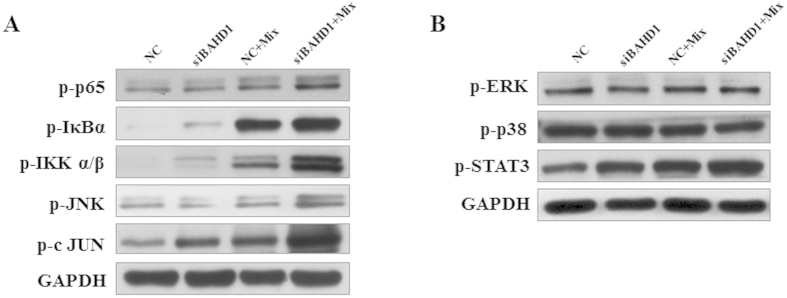
Activation of the IKK/NF-κ B and JNK/AP-1 pathways, but not the ERK1/2 and p38 pathways, in siBAHD1-treated Caco-2 cells. Caco-2 cells were treated with negative control siRNA or BAHD1 siRNA. At 48 h, they were exposed to the stimulating mixture (inflammatory mediators including TNF-α, IFN-γ, IL-1β and LPS described before) for 5 minutes (NC+Mix group, siBAHD1+Mix group) or not (NC group, siBAHD1 group). (**A**) The siBAHD1 group showed stronger activation of key proteins in the NF-κ B (the level of p-IKK α/β, p-IκBα, p-p65 increased) and JNK/AP-1 (phosphorylation level of JNK and c-JUN enhanced) pathways. (**B**) The phosphorylation level of STAT3 showed the same trend as in (**A**). Other MAPK pathways such as P38 and ERK1/2 did not show a significant difference between the NC and siBAHD1 groups in the inflammatory environment. Representative western blots of cell lysates are shown from at least three independent experiments.

In response to stimulation by the various factors described above, activity through two important inflammation pathways, the IKK/NF-κ B and JNK/AP-1 pathways, was measured by using relevant key phospho-specific antibodies to investigate the underlying molecular mechanism of BAHD1 deficiency in the cell model. Activation was comparable in the stimulation only group and the siBAHD1 group. *In vitro*, siBAHD1 in Caco-2 cells resulted in stronger phosphorylation of IKK α/β, IκBα and NF-κ B subunit p65 in the IKK/NF-κ B pathway. Moreover, the AP-1 protein c-JUN and upstream regulator JNK showed higher phosphorylation levels in the siRNA-treated group [Fig. 5A]. Regarding other MAPK pathways active during the acute phase in cell injury, we did not observe activation of either the ERK1/2 pathway or the p38 pathway in the siBAHD1 group [Fig. 5B]. By contrast, STAT3 phosphorylation was clearly increased, indicating that there might some connection between BAHD1 and the Janus kinase/signal transducer and activator of transcription (JAK/STAT) pathway [Fig. 5B]. These results were in accordance with the enhanced levels of cytokines in the siRNA-treated group, indicating that BAHD1 might be associated with cytokine expression in intestinal cells through a certain inflammatory pathway.

### An NF-κ B inhibitor Blocked the Effects caused by BAHD1 Repression in Caco-2 Cell model

**Figure 6:**
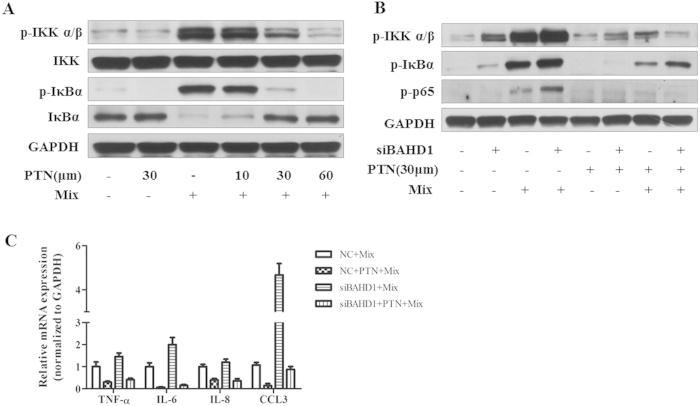
An NF-κ B inhibitor blocked the effects caused by BAHD1 repression in Caco-2 cells. Caco-2 cells were pre-treated with PTN before exposure to the inflammatory mediators mixture (Mix). (**A**) PTN inhibited the activation of the NF-kB pathway: the phosphorylation of IKK α/β and IκBα decreased significantly at a concentration of 30 μm with little effect on cell viability [Supplementary Fig. S6]. (**B**) The NF-κ B inhibitor PTN eliminated the phosphorylation difference of IKK, IκBα and NF-κ B p65 in the NC/siBAHD1-treated cell model. (**C**) PTN repressed the expression of associated inflammatory genes such as TNF-α, IL-6, IL-8 and CCL3 *in vitro*. The values represent the mean ± SEM of three independent experiments. Representative blots of cell lysates are shown from at least three independent experiments.

We had found that IKK and IκBα were activated in the cell model system; therefore, we wondered whether its downstream target, NF-κ B, participated in the regulation of cytokine gene expression. To confirm that the NF-κ B pathway was involved in the regulation by BAHD1 of inflammatory gene expression in the cell model, we used an NF-κ B inhibitor, parthenolide (PTN, 30 μm), which is a sesquiterpene lactone that inhibits activation of the NF-κ B pathway^15^. It significantly inhibited the stimulus-induced activation of the NF-κ B pathway and repressed the expression of associated inflammatory genes [Fig. 6A,C]. After treatment with siRNA for 48 h, Caco-2 cells were incubated with PTN for 1 hour before the addition of the mixture of various stimulators described above. The phosphorylation levels of IKKα/β, IκBα and NF-κ B p65 were reduced significantly in both the NC and siBAHD1 groups [Fig. 6B], which was consistent with a decrease in cytokines such as TNF-α, IL-6, IL-8, CCL3 [Fig. 6C]. Therefore, the results suggested that cytokines were highly activated mainly through the NF-κ B pathway.

### BAHD1 Differentially Modulated the TNF Signalling Pathway by Altering TNFR1 Expression

**Figure 7:**
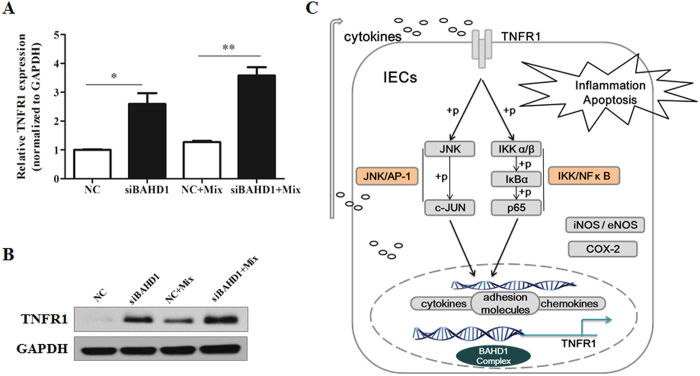
BAHD1 modulated the TNF signalling pathway by altering TNFR1 expression. Caco-2 cells were incubated with BAHD1 siRNA (siBAHD1) and a negative control siRNA (NC) before incubation with the mixture (Mix) for 24 h to mimic gut inflammation. (**A**) mRNA and (**B**) protein levels showed significantly increased contents of TNFR1 in the siBAHD1 group compared with the NC group. (**C**) A hypothesis regarding BAHD1 negative regulation of the TNF signalling pathway. The data are expressed as the mean ± SEM; n = 3, *p < 0.05; **p < 0.001 versus NC group. For the blots, independent experiments were repeated at least three times.

Downregulation of BAHD1 correlated positively with cytokine secretion; therefore, we turned our attention to the starting point of this secretory process. The NF-κ B and AP-1 pathways, which are critical for expression of the proinflammatory cytokine cascade, might be mediated by tumour necrosis factor receptor 1 (TNFR1)^16^. Consistent with the high activation of key proteins in intracellular cell signalling pathways, we detected that the expression of TNFR1 increased dramatically in the siBAHD1 group, at both the protein and mRNA levels, compared with the negative control group [Fig. 7A,B]. High levels of cytokines secreted by colonic epithelium that had lost BAHD1 expression might in turn result in persistent activation of the NF-κ B and JNK/AP-1 pathways. Taken together, these results suggest an essential role of BAHD1 in the negative regulation of the starting point of the pathway through the TNF signalling pathway.

## Discussion

Here, we used a systematic computational approach based on publicly available gene expression signatures to predict multiple previously undescribed molecules for UC^10^. Distance values derived from comparing public UC microarray data against a compendium of gene expression signatures comprising 106 siRNAs were evaluated in the datasets based on GSEA. All siRNAs were manually filtered according to their quality from a whole range of data in the GEO platform. A small distance indicates a similar gene regulation tendency, and among the smallest-scoring siRNAs predicted from our approach were EZH2, UPF1, FOXM1, NUDT6 and BAHD1.

The exact functions of these five factors in IBD have not been previously reported. EZH2 (enhancer of zeste homolog 2) is a histone methyltransferase associated with transcriptional repression, and its overexpression promotes tumour development^17^. UPF1 (up-frameshift mutant 1) is the regulator of nonsense transcripts 1 in humans and participates in both nuclear mRNA export and mRNA surveillance^18^. The third one, FOXM1, is a transcription factor involved in cell cycle progression that regulates the expression of a large number of G2/M-specific genes^19^, while NUDT6 (nucleoside diphosphate-linked moiety X motif 6) is thought to be a fibroblast growth factor antisense gene associated with cell cycle progression and tumour proliferation^20^. Further study into the potential genes driving this clustering could reveal new information regarding UC’s pathogenesis and new molecular therapeutic directions.

Among them, BAHD1 is involved in gene silencing^21^ and was the most downregulated in the UC mouse model, which was inferred to indicate the most potential as a regulatory protein for the disease. The experimental validation we performed *in vitro* confirmed that the loss of BAHD1 activated various cytokines during a cellular immune response through associated signalling pathways. Intestinal inflammation and tissue damage is a direct result of increased circulating inflammatory cytokines, which are secreted at sites of inflammation and impact during the onset, progression, and resolution of UC^22^. Those cytokines, and also COX-2, iNOS and eNOS, are mediated by several signalling pathways^23^,^24^.

Transcription factors, including NF-κ B and AP-1, play critical roles in the expression of genes involved in inflammation and carcinoma development in the gastrointestinal tract^25^,^26^. In the present study, we showed that key proteins in the NF-κ B pathway, including IKK α/β, IκBα and NF-κ B subunit p65^27^,^28^, were activated to a higher level in stimulated Caco-2 cells with BAHD1 knocked down compared with a purely stimulated group. AP-1 is a member of a family of transcription factors mainly belonging to the JUN and Fos families whose activation is involved in inflammatory gene expression^29^. A similar phenomenon was observed in the JNK/AP-1 pathway, in which the phosphorylation levels of JNK and c-JUN increased. Taken together, the data gave the strongest hint that a link might exist between activation of the transcription factors NF-κ B and AP-1 and the reduction in BAHD1 expression in IECs.

Pathogen-associated molecular patterns are sensed by specific receptors, which in turn activate signalling cascades to induce the synthesis of inflammatory mediators such as TNF, IL-1 and IFN^30^. TNFR1, which is ubiquitously expressed, has pleiotropic functions related to cell immunity, survival, apoptosis and necrosis and can be activated via both membrane-bound and soluble TNF^31^,^32^. The TNF receptor is primarily responsible for initiating inflammatory responses by mediating TNF-α- induced NF-κ B activation^33^,^34^. In this study, TNFR1 transcription increased significantly in Caco-2 cells after downregulation of BAHD1, causing TNF signalling pathway activation in IECs during inflammatory mediator exposure. As a result, more cytotoxic inflammatory factors were produced in response to the activation of the pathway, which in turn resulted in inflammatory status aggravation with more secreted cytokines, especially TNF combined with TNFR1. The continuous excessive cytokine secretion caused injury and dysfunction of the IECs. A hypothesis of BAHD1 negatively regulating the TNF signalling pathway by altering TNFR1 expression is shown in Fig. 7C, in which the inflammatory microenvironment induces downregulation of BAHD1 in IECs, which in turn can increase the production of various cytokines through the IKK/NF-κ B and JNK/AP-1 pathway.

As a novel heterochromatinization factor in vertebrates, BAHD1 participates in gene silencing by promoting the formation of heterochromatin through interaction with HP1, MBD1, HDAC5 and several transcription factors to control cell differentiation and maintenance of homeostasis^21^. We speculate that in some way, BAHD1 connects with other repressive core complex factors to mediate TNFR1 gene silencing.

As for the MAPK pathways, the JNK pathway and the p38 MAPK pathway regulate apoptotic cell death, whereas ERK1/2 acts as a prosurvival factor that contributes to the regulation of cell proliferation and differentiation^35^. Therefore, JNK activation in IECs with downregulated BAHD1 levels during the acute phase of injury may result directly in apoptosis, not survival.

The JAK/STAT pathway is a central mediator of the responses of various extracellular cytokines and has been implicated in the pathogenesis of many human immunity disorders, including IBD. JAK inhibitors have the potential to treat the inflammation associated with colitis^36^,^37^. The detailed mechanism whereby repression of BAHD1 in enteric cells leads to higher levels of STAT3 phosphorylation requires further exploration.

Although we found some interesting results, it should be noted that follow-up investigative work will be necessary. First, *in vivo* evidence regarding the precise role of BAHD1 in UC is lacking. In addition, although exposure of Caco-2 cells to inflammatory stimuli is a common approach to investigating inflammatory signalling, it is not the disease per se. Finally, the detailed mechanism of how BAHD1 represses TNF receptor expression is unclear and will require deep exploration.

In conclusion, the results raise the intriguing possibility that a computationally predicted protein, BAHD1, might act as an important regulator of the classical inflammation pathways in UC, and there might be a functional association between intestinal cell inflammatory responses and BAHD1. Additionally, the computational approaches we used based on public gene expression databases show potential for the discovery of genes and proteins that might be vital factors in the pathogenesis of certain diseases.

## Methods

### Human Tissue

Human paraffin-embedded colonic mucosa pinches were obtained from surgical patients with active UC or healthy adjacent or distant colonic tissue from subjects with certain cancers. Pathological analysis verified the diagnoses of UC. This study was approved by the Ethics Committee of the First Affiliated Hospital, College of Medicine, Zhejiang University, Hangzhou, China. Informed consent was obtained from each subject before the study. The study protocols and the consent form were administered in accordance with the approved guidelines of the Ethics Committee.

### Animal Treatment

Six- to eight-week-old littermate female C57BL/6 mice were purchased from Zhejiang Experimental Animal Centre, Hangzhou, China. Mice were housed in an animal room with air-conditioned specific pathogen-free (SPF) conditions at 23 ± 2 °C with a 12 h light/dark cycle, and they were acclimated for 7 days before experimentation. The Animal Care and Use Committee of Zhejiang University approved all the mouse studies, which were performed in accordance with the Chinese guidelines for the care and use of laboratory animals.

### Computation of Distances between Different Expression Data

UC patient tissue expression profiles and siRNA-perturbed cell expression profiles were collected from the National Center for Biotechnology Information (NCBI) Gene Expression Omnibus (GEO)^38^. To make full use of collected expression data, the data platforms were restricted to the Affymetrix Human Genome U133A Array (GPL96) and the Affymetrix Human Genome U133 Plus 2.0 Array (GPL570), the most widely used *Homo sapiens* platform. Any probes not numbered in all the datasets were excluded in our next analysis, resulting in 22,215 validated probes. Expression profiles representing different siRNA permutations were collected by searching in the GEO with the key words siRNA, shRNA and by manual checking. Due to the dearth of siRNA perturbation expression profiles, the cell types used were not restricted to particular cell types [Supplementary Table S3].

To properly compare expression profiles from different datasets, the validated probes were ranked by their expression change compared to the control as described below.

First, we paired each experiment’s (UC patient samples or siRNA processed cells) expression profile to a control (healthy controls or untreated cells).

Second, for each pair of samples, we ranked the probes considering both their fold-changes and their absolute values relative to a probe rank list (PRL)^11^. The detailed steps are list below.

(1) Expression values less than the primary threshold value (the lower quartile of expression values of these two samples) were set to that value. (2) The probes were then ranked in descending order of corresponding experiment-to-control ratio values. (3) For probes where the ratio value equals one, the secondary threshold value (one-tenth of the primary threshold value) was used to reset the value of these probes. (4) These probes were sub-sorted in descending order of the new ratios to produce the final probe rank list for each pair of samples. 

This hierarchical sort strategy avoids the inappropriately high or low ranks that can be caused by large fold changes resulting from dividing by small values.

Third, PRLs representing the same permutation (same disease or same gene silencing) were combined with the R package GeneExpressionSignature^39^ into final PRLs to represent the cell’s responses to them according to a hierarchical majority-voting scheme^13^,^40^.

Then, the distance between UC and the different siRNA perturbations was estimated by GSEA^10^ in the following steps:

 (1) The distances were calculated by comparing two PRLs through investigating whether the top- (or bottom-) ranked probes in one PRL was also the top- (or bottom-) ranked in the other PRL. Thus, for each PRL, a gene set containing its top- (or bottom-) ranked probes was generated as its signature. The top and bottom 250 probes were selected as the signature of each PRL. The sizes of the signatures were changeable, and their effect on the final prediction was limited; therefore, the size was set to an empirical value from a reference paper^41^. (2) When comparing two PRLs A and B, the enrichment score of A’s (or B’s) signature in reference PRL B (or A) was calculated by equal weighted GSEA^10^,^11^, and the enrichment score could be presented as ES_AB_(or ES_BA_) ranging from −1 to 1. A high enrichment score indicates that the signature genes also tend to appear at the top or bottom of the reference PRL, indicating similarity between them. (3) The distance between each two PRLs was defined as 1-(ES_AB_+ES_BA_)/2 ranging from 0 to 2.

### Immunohistochemistry (IHC)

Colon sections (4 μm) cut from paraffin-embedded intestine tissues were deparaffinized with xylene and rehydrated with ethanol. For IHC, tissue sections were preincubated with 10% normal goat serum (ZSGB-BIO, Beijing, China) in PBS (pH 7.5) and then incubated with primary antibodies against BAHD1 (dilution: 1:200, Abcam, Cambridge, UK) overnight at 4 °C. Tissue sections were stained with HPR secondary antibody (dilution: 1:1000, ZSGB-BIO, Beijing, China) for 1 h at 37 °C in an incubator. Immunoreactivity was detected using a DAB kit (ZSGB-BIO, Beijing, China) and visualized as brown staining.

### Cell Culture

The heterogeneous human epithelial colorectal adenocarcinoma cell line, Caco-2 (Institute of Biochemistry and Cell Biology, China Academy of Sciences, Shanghai, China) was maintained in Minimum Essential Medium (MEM, Invitrogen, Carlsbad, CA, USA), supplemented with 10% fetal calf serum (FCS, Invitrogen, Carlsbad, CA, USA) and 100 U/ml penicillin-streptomycin (Sigma-Aldrich, St. Louis, MO, USA) at 37 °C in a humidified 5% CO2 atmosphere.

### RNA Mediated Interference

Small-interfering RNA (siRNA)-mediated knockdown in human Caco-2 cells was performed using negative control (NC) siRNA (Invitrogen, Carlsbad, CA, USA), and a specifically designed siRNA with the sequence 5′-AUA GCA CUU CUC CUC AAU GCA GGC C-3′ to target human BAHD1. Lipofectamine 2000 (Invitrogen, Carlsbad, CA, USA) was used as the siRNA transfection reagent, as per the manufacturer’s instructions. siRNAs were used at a final concentration of 15 nM. The interfering effect was verified by qPCR and western blotting for the mRNA or protein, respectively [Supplementary Fig. S4].

### Cytokines Measurement

To evaluate proinflammatory factors in the DSS-induced colitis in mice, distal colons cut longitudinally were washed in phosphate buffered saline (PBS). Strips of 100 mg of colon tissue were placed in clean EP tubes containing 0.5 ml cold PBS and ground with a pestle. The samples were centrifuged at 14,000 g for 10 minutes at 4 °C. The supernatant was collected to quantify the production of TNF-α, IL-6 and IFN-γ by enzyme-linked immunosorbent assay (ELISA) kits (eBioscience, San Diego, CA, USA), according to the manufacturer’s protocol. Similarly, for cell culture, cell-free supernatant was harvested after treatment and analyzed for IL-6, IL-8 and MCP-1 contents.

### Gene Expression Measure

RNAiso Plus (Takara, Otsu, Japan) was used to isolate mRNA from cells or tissue. The PrimeScript RT Master Mix (Takara, Otsu, Japan) was used to generate cDNA. qPCR was performed using a SYBR Green Premix DimerEraser (Takara, Otsu, Japan) on the 7900HT Fast Real-Time PCR system (Applied Biosystems). All reactions had a melting curve with a single peak. The cycle threshold (CT) values for the triplicate samples were averaged and the data were analyzed using the ΔΔCT method, where fold change = 2^−ΔΔCT^. All data analyzed were normalized to beta-Actin or GAPDH expression. For the specific primer sequences (Sangon Biotech, Shanghai, China), see Supplementary Table S4.

### Western Blot Analysis

To detect the expression of associated proteins, Caco-2 cells and distal colon tissue of mice were washed with cold PBS, collected and lysed in RIPA buffer (Pulilai BioTech, Beijing, China) containing protease inhibitor cocktail (Sigma-Aldrich, St. Louis, MO, USA). Proteins (30 μg/sample) were separated by SDS/PAGE and transferred to a polyvinylidene difluoride membrane (0.45 mm pore; Millipore, Bedford, MA, USA). After being blocked with 5% skim milk powder diluted in TBS containing 5% Tween-20 for 1 h, the membrane was incubated with a primary antibody, anti-p-NF κB (S536), p-IKK-alpha (S176)/IKK-beta (S177), anti-p-IκB-alpha, anti-p-c-JUN (S73), anti-p-STAT3 (Y705), anti-TNF-R1, anti-GAPDH (Cell Signaling Technology, Danvers, MA, USA); anti-p-p38 (Y182+T180) anti-BAHD1 (Abcam, Cambridge, UK); or anti-JNK1 (pY185)/JNK2 (pY185)/JNK3 (pY223) (Epitomics, Burlingame, CA, USA), at 4 °C overnight. Immunoreactive proteins were detected using an enhanced chemiluminescence light (ECL) detecting kit (Lianke Multi Sciences, Hangzhou, China). GAPDH acted as a loading control. The experiments were replicated at least three times, and representative results are shown.

### Establishment of DSS-induced Colitis in Mice

Chemically induced murine models of intestinal inflammation are the most commonly used and best-described models for investigating the pathophysiological mechanisms and immunological processes underlying chronic mucosal inflammation^42^. The protocol to develop acute colitis in C57BL/6 mice was as described previously^43^. The drinking supply of the mouse cages was filled with DSS (MW: 36,000–50,000; MP Biochemicals, Solon, OH, USA) at 3% weight/volume, while control mice received autoclaved water for 7 days. Mice receiving DSS orally developed acute UC-like clinical and pathological manifestations. The mice developed colonic mucosal inflammation limited to the mucosa and contained large numbers of immunoglobulin-secreting plasma cells, accompanied by body weight loss, bloody diarrhoea during the acute phase, and shortening of the colon. In accord with these manifestations, the inflamed colon tissue secreted much more TNF-α, IL-6 and IFN-γ. [Supplementary Fig. S5] Disease activity index (see Supplementary Materials) and histological analysis of haematoxylin & eosin (H&E)-stained colon sections [Supplementary Table S5] were the two main standards that identified the successful establishment of the UC-like mouse model.

### Establishment of a Cell Model to Simulate Inflammatory Environment for IECs

**Figure 8:**
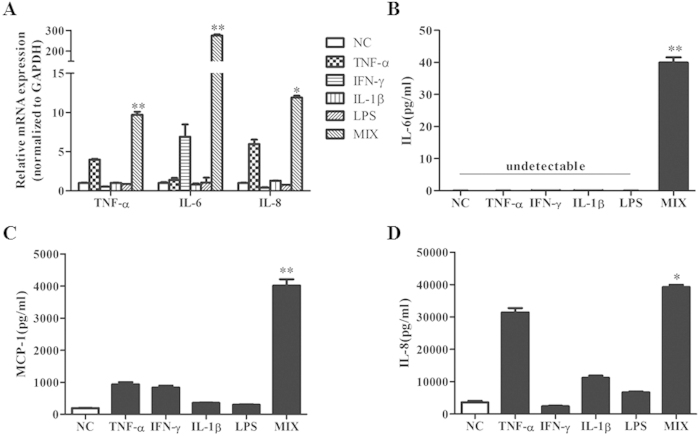
Establishment of a cell model to simulate the inflammatory environment of IECs. An *in vitro* model to estimate gut inflammation using various proinflammatory factors including TNF-α, IFN-γ and IL-1β, and the gram-negative bacterial product LPS. Caco-2 cells were incubated with each factor separately and with their mixture (MIX) for 24 hours. RNA was extracted and qPCR was performed. (**A**) qPCR analysis of TNF-α, IL-6 and IL-8 expression. The data were normalized to the expression of GAPDH. (**B**)(**C**)(**D**) Stimulated production of IL-6, MCP-1 and IL-8 in culture supernatant of Caco-2 cells in different exposure groups measured using ELISA kits. The values are expressed as the mean ± SEM of three separate experiments performed in triplicate. *P < 0.05, **P < 0.01. The increase in the MIX group was more significant than in any of the other stimulated groups after 24 h of exposure.

To develop an vitro model to investigate inflammatory signalling in IECs, Caco-2 cells were exposed to the inflammatory mediators LPS (1 μg/ml) (Sigma-Aldrich, St. Louis, MO, USA), tumour necrosis factor-α (rh TNF-α, 50 ng/ml), recombinant human interferon-γ (rh IFN-γ, 50 ng/ml), and interleukin-1 beta (rh IL-1β, 25 ng/ml) (Peprotech, Rocky Hill, NJ, USA) for 24 h^44^. Using qPCR and ELISA, we found that exposure to a mixture of these factors together (MIX) in Caco-2 cells for 24 h mimicked closely the environment of gut inflammation as the cells expressed the highest amounts of proinflammatory factors (TNF-α, IL-6) and chemokines (IL-8, MCP-1) [Fig. 8].

### Statistical Analysis

All data are shown as the mean ± SEM or SD value from at least three independent experiments. Significant differences were evaluated by the unpaired Student’s *t*-test with two-tailed distributions. P-values below 0.05 were considered significant. The results were considered significant at *P < 0.05; **P < 0.01. Prism version 5.0 (Graph Pad Software) was used to perform the statistical analyses.

## Additional Information

**How to cite this article**: Zhu, H. *et al.* Computational Prediction and Validation of BAHD1 as a Novel Molecule for Ulcerative Colitis. *Sci. Rep.* **5**, 12227; doi: 10.1038/srep12227 (2015).

## Supplementary Material

Supplementary Information

## References

[b1] Ordas I., Eckmann L., Talamini M., Baumgart D. C. & Sandborn W. J. Ulcerative colitis. Lancet. 380, 1606–1619 (2012). 22914296 10.1016/S0140-6736(12)60150-0

[b2] Ng S. C. Epidemiology of inflammatory bowel disease: Focus on Asia. Best. Pract. Res. Cl. Ga. 28, 363–372 (2014). 10.1016/j.bpg.2014.04.003 24913377

[b3] Molodecky N. A. *et al.* Increasing Incidence and Prevalence of the Inflammatory Bowel Diseases With Time, Based on Systematic Review. Gastroenterology. 142, 46–54 (2012). 22001864 10.1053/j.gastro.2011.10.001

[b4] Wirtz S. & Neurath M. F. Mouse models of inflammatory bowel disease. Adv. Drug. Deliver. Rev. 59, 1073–1083 (2007). 10.1016/j.addr.2007.07.003 17825455

[b5] Sartor R. B. Mechanisms of disease: pathogenesis of Crohn’s disease and ulcerative colitis. Nat. Clin. Pract. Gastr. 3, 390–407 (2006). 10.1038/ncpgasthep0528 16819502

[b6] Smale S. T. Selective transcription in response to an inflammatory stimulus. Cell. 140, 833–844 (2010). 20303874 10.1016/j.cell.2010.01.037 PMC2847629

[b7] Feng Y. J. & Li Y. Y. The role of p38 mitogen-activated protein kinase in the pathogenesis of inflammatory bowel disease. J. Digest. Dis. 12, 327–332 (2011). 10.1111/j.1751-2980.2011.00525.x 21955425

[b8] Schena M., Shalon D., Davis R. W. & Brown P. O. Quantitative monitoring of gene expression patterns with a complementary DNA microarray. Science. 270, 467–470 (1995). 7569999 10.1126/science.270.5235.467

[b9] Warner E. E. & Dieckgraefe B. K. Application of genome-wide gene expression profiling by high-density DNA arrays to the treatment and study of inflammatory bowel disease. Inflamm. Bowel. Dis. 8, 140–157 (2002). 11854614 10.1097/00054725-200203000-00012

[b10] Subramanian A. *et al.* Gene set enrichment analysis: a knowledge-based approach for interpreting genome-wide expression profiles. P. Natl. Acad. Sci. USA. 102, 15545–15550 (2005). 10.1073/pnas.0506580102 PMC1239896 16199517

[b11] Lamb J. *et al.* The connectivity map: Using gene-expression signatures to connect small molecules, genes, and disease. Science. 313, 1929–1935 (2006). 17008526 10.1126/science.1132939

[b12] Dudley J. T. *et al.* Computational Repositioning of the Anticonvulsant Topiramate for Inflammatory Bowel Disease. Sci. Transl. Med. 3 10.1126/scitranslmed.3002648 (2011). PMC3479650 21849664

[b13] Iorio F. *et al.* Discovery of drug mode of action and drug repositioning from transcriptional responses. P. Natl. Acad. Sci. USA. 107, 14621–14626 (2010). 10.1073/pnas.1000138107 PMC2930479 20679242

[b14] Panes J. Adhesion molecules: their role in physiopathology and treatment of inflammatory bowel disease. Gastroenterol. Hepatol. 22, 514–524 (1999). 10650667

[b15] Sheehan M. *et al.* Parthenolide, an inhibitor of the nuclear factor-kappa B pathway, ameliorates cardiovascular derangement and outcome in endotoxic shock in rodents. Mol. Pharmacol. 61, 953–963 (2002). 11961112 10.1124/mol.61.5.953

[b16] Chu W. M. Tumor necrosis factor. Cancer letters. 328, 222–225 (2013). 23085193 10.1016/j.canlet.2012.10.014 PMC3732748

[b17] Vire E. *et al.* The Polycomb group protein EZH2 directly controls DNA methylation. Nature. 439, 871–874 (2006). 16357870 10.1038/nature04431

[b18] Cheng Z. H., Muhlrad D., Lim M. K., Parker R. & Song H. W. Structural and functional insights into the human Upf1 helicase core. Embo. J. 26, 253–264 (2007). 17159905 10.1038/sj.emboj.7601464 PMC1782376

[b19] Wierstra I. & Alves J. FOXM1, a typical proliferation-associated transcription factor. Biol. Chem. 388, 1257–1274 (2007). 18020943 10.1515/BC.2007.159

[b20] Baguma-Nibasheka M., Macfarlane L. A. & Murphy P. R. Regulation of fibroblast growth factor-2 expression and cell cycle progression by an endogenous antisense RNA. Genes. 3, 505–520 (2012). 24704982 10.3390/genes3030505 PMC3899992

[b21] Bierne H. *et al.* Human BAHD1 promotes heterochromatic gene silencing. P. Natl. Acad. Sci. USA. 106, 13826–13831 (2009). 10.1073/pnas.0901259106 PMC2728979 19666599

[b22] Christophi G. P., Rong R., Holtzapple P. G., Massa P. T. & Landas S. K. Immune markers and differential signaling networks in ulcerative colitis and Crohn’s disease. Inflamm. Bowel. Dis. 18, 2342–2356 (2012). 22467146 10.1002/ibd.22957 PMC3407828

[b23] Hobbs S. S. *et al.* TNF transactivation of EGFR stimulates cytoprotective COX-2 expression in gastrointestinal epithelial cells. Am. J. Physiol-Gastr. L. 301, G220–229 (2011). 10.1152/ajpgi.00383.2010 PMC3154604 21566012

[b24] Pelletier J. P. & Martel-Pelletier J. The Novartis-ILAR rheumatology prize 2001 osteoarthritis: from molecule to man. Arthritis. Res. 4, 13–19 (2002). 11879533 10.1186/ar378 PMC128913

[b25] Schmid R. M., Adler G. & Liptay S. Activation of NF kappaB in inflammatory bowel disease. Gut. 43, 587–588 (1998). 9882195 10.1136/gut.43.4.586c PMC1727295

[b26] Ben-Neriah Y. & Karin M. Inflammation meets cancer, with NF-kappa B as the matchmaker. Nat. Immunol. 12, 715–723 (2011). 21772280 10.1038/ni.2060

[b27] Hayden M. S. & Ghosh S. NF-kappaB, the first quarter-century: remarkable progress and outstanding questions. Gene. Dev. 26, 203–234 (2012). 22302935 10.1101/gad.183434.111 PMC3278889

[b28] Smale S. T. Hierarchies of NF-kappaB target-gene regulation. Nat. Immunol. 12, 689–694 (2011). 21772277 10.1038/ni.2070 PMC3169328

[b29] Schonthaler H. B., Guinea-Viniegra J. & Wagner E. F. Targeting inflammation by modulating the Jun/AP-1 pathway. Ann. Rheum. Dis. 70, I109–I112 (2011). 21339212 10.1136/ard.2010.140533

[b30] Gaestel M., Kotlyarov A. & Kracht M. Targeting innate immunity protein kinase signalling in inflammation. Nat. Rev. Drug. Discov. 8, 480–499 (2009). 19483709 10.1038/nrd2829

[b31] Varfolomeev E. *et al.* Cellular inhibitors of apoptosis are global regulators of NF-kappaB and MAPK activation by members of the TNF family of receptors. Sci Signal. 5, 10.1126/scisignal.2001878 (2012). 22434933

[b32] Naude P. J. W., den Boer J. A., Luiten P. G. M. & Eisel U. L. M. Tumor necrosis factor receptor cross-talk. Febs. J. 278, 888–898 (2011). 21232019 10.1111/j.1742-4658.2011.08017.x

[b33] Loetscher H., Stueber D., Banner D., Mackay F. & Lesslauer W. Human Tumor-Necrosis-Factor-Alpha (Tnf-Alpha) Mutants with Exclusive Specificity for the 55-Kda or 75-Kda Tnf Receptors. J. Biol. Chem. 268, 26350–26357 (1993). 8253759

[b34] Chen G. & Goeddel D. V. TNF-R1 signaling: a beautiful pathway. Science. 296, 1634–1635 (2002). 12040173 10.1126/science.1071924

[b35] Xia Z. G., Dickens M., Raingeaud J., Davis R. J. & Greenberg M. E. Opposing Effects of Erk and Jnk-P38 Map Kinases on Apoptosis. Science. 270, 1326–1331 (1995). 7481820 10.1126/science.270.5240.1326

[b36] Coskun M., Salem M., Pedersen J. & Nielsen O. H. Involvement of JAK/STAT signaling in the pathogenesis of inflammatory bowel disease. Pharmacol. Res. 76, 1–8 (2013). 23827161 10.1016/j.phrs.2013.06.007

[b37] Sandborn W. J. *et al.* Phase 2 Randomized Study of CP-690,550, an Oral Janus Kinase Inhibitor, in Active Crohn’s Disease. Gastroenterology. 140, S124–S124 (2011).

[b38] Iorio F., Tagliaferri R. & di Bernardo D. Identifying network of drug mode of action by gene expression profiling. J. Comput. Biol. 16, 241–251 (2009). 19183001 10.1089/cmb.2008.10TT

[b39] Li F. *et al.* GeneExpressionSignature: an R package for discovering functional connections using gene expression signatures. Omics. 17, 116–118 (2013). 23374109 10.1089/omi.2012.0087

[b40] Ni M. *et al.* ExpTreeDB: Web-based query and visualization of manually annotated gene expression profiling experiments of human and mouse from GEO. Bioinformatics. 30, 3379–3386 (2014). 25152233 10.1093/bioinformatics/btu560

[b41] Burczynski M. E. *et al.* Molecular classification of Crohn’s disease and ulcerative colitis patients using transcriptional profiles in peripheral blood mononuclear cells. J. Mol. Diagn. 8, 51–61 (2006). 16436634 10.2353/jmoldx.2006.050079 PMC1867573

[b42] Alex P. *et al.* Distinct Cytokine Patterns Identified from Multiplex Profiles of Murine DSS and TNBS-induced Colitis. Inflamm. Bowel. Dis. 15, 341–352 (2009). 18942757 10.1002/ibd.20753 PMC2643312

[b43] Wirtz S., Neufert C., Weigmann B. & Neurath M. F. Chemically induced mouse models of intestinal inflammation. Nat. Protoc. 2, 541–546 (2007). 17406617 10.1038/nprot.2007.41

[b44] Van De Walle J., Hendrickx A., Romier B., Larondelle Y. & Schneider Y. J. Inflammatory parameters in Caco-2 cells: Effect of stimuli nature, concentration, combination and cell differentiation. Toxicol. In. Vitro. 24, 1441–1449 (2010). 20406675 10.1016/j.tiv.2010.04.002

