# Supplementary material for: Computational Prediction and Validation of BAHD1 as a Novel Molecule for Ulcerative Colitis
Source: Sci Rep. 2015 Jul 17;5:12227. doi: 10.1038/srep12227 (PMC4505333; doi:10.1038/srep12227)
Supplement: Supplementary Information [file srep12227-s1.doc]

# Supplementary Materials

**Computational Prediction and Validation of BAHD1 as a Novel Molecule for Ulcerative Colitis**

Huatuo Zhu^1,*^, Xingyong Wan^1,*^, Jing Li^2,*^, Lu Han^3^, Xiaochen Bo^3^, Wenguo Chen^1^, Chao Lu^1^, Zhe Shen^1^, Chenfu Xu^1^, Lihua Chen^1,#^, Chaohui Yu^1,#^ & Guoqiang Xu^1,#^

**Supplementary Figures and Legends**

**
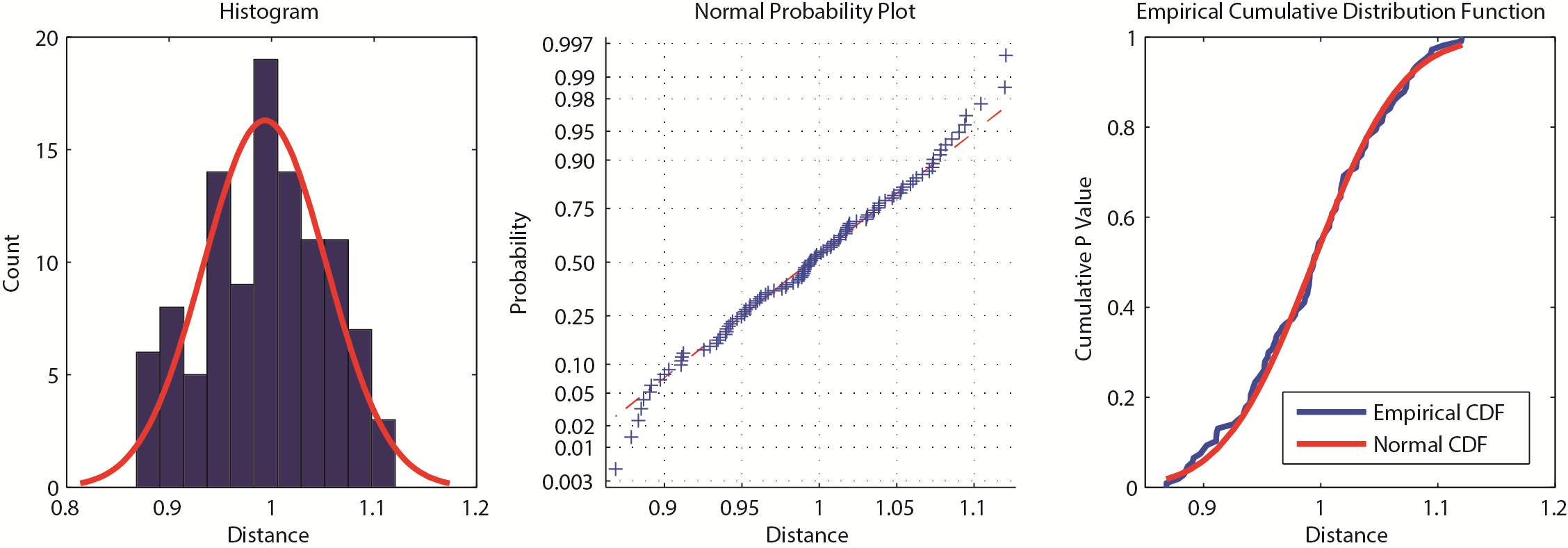
**

**Figure S1.** Histogram, normal probability plot and empirical cumulative distribution function of distances between UC phenotype and siRNA perturbations. The distances between UC phenotype and SiRNA perturbations form a normal distribution (tested by one-sample Kolmogorov-Smirnov test at the 5% significance level) with μ=0.994 and σ=0.060.


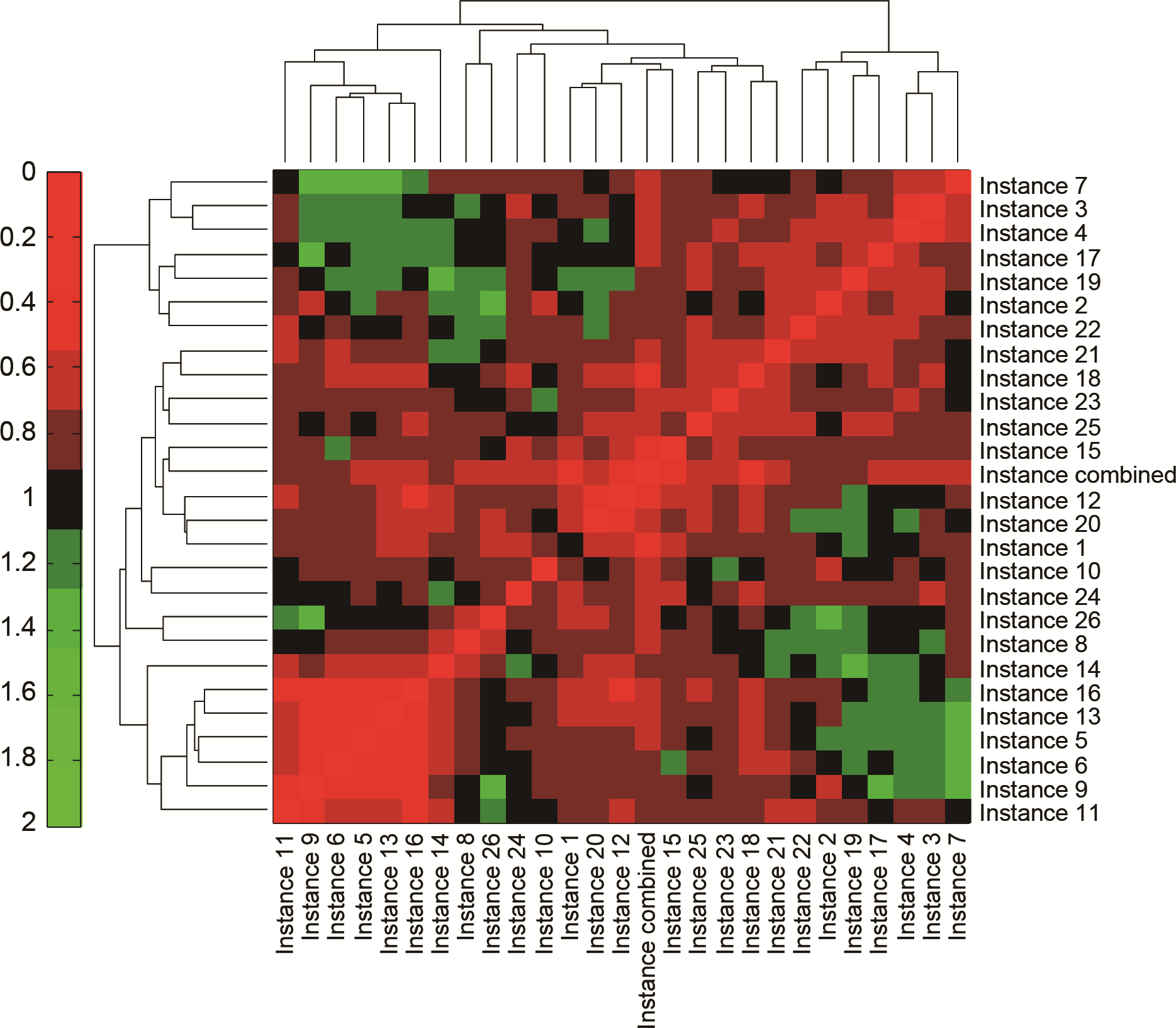


**Figure S2.** Clustergram of distances between 26 ulcerative colitis instances and the combined one (marked as instances combined). An instance contains a pair of samples collected from a patient and a healthy individual. The information regarding each instance can be found in Table S2. The combined instance was generated by the R package GeneExpressionSignature. The average distance between different instances is 0.854, and the average distance from the 26 individual instances to the combined one is 0.662.

**
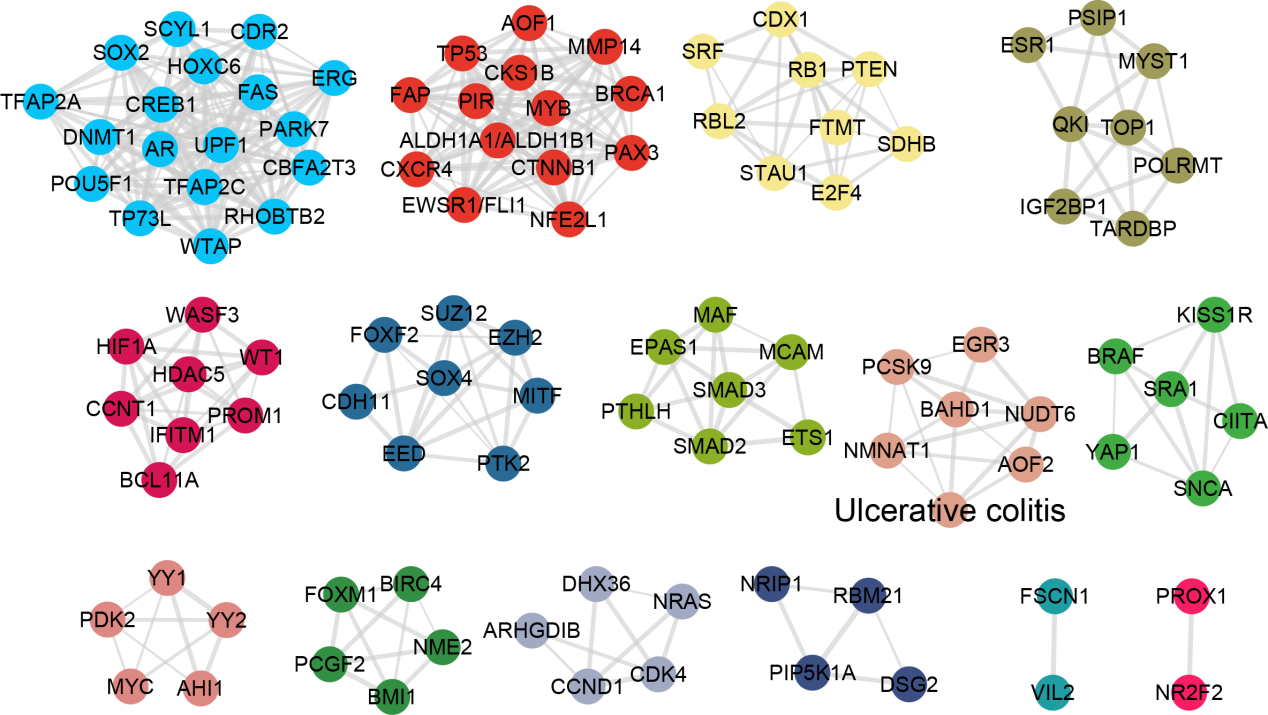
**

**Figure S3.** Cluster results of ulcerative colitis and siRNA perturbations performed by affinity propagation clustering algorithm^1^, a parameter-free clustering algorithm widely used in multiple studies using GSEA to explore biological relations^2,3,4^.

**
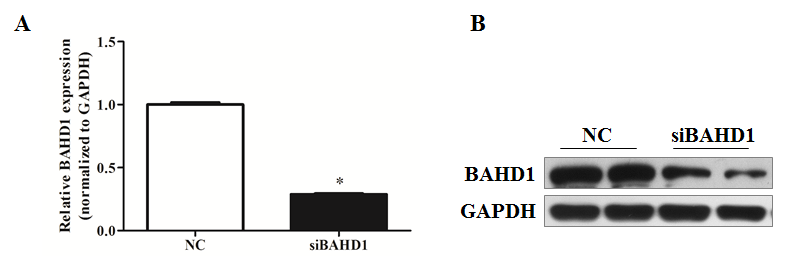
**

**Figure S4.** siRNA-mediated silencing of BAHD1 expression in Caco-2 cells compared with negative control siRNA-transfected cells (NC). Caco-2 cells were treated with siBAHD1 for 48 h, and then replaced with fresh medium for 24 h. (A) qPCR analysis and (B) western blot results showed that BAHD1 expression was significantly decreased in the siBAHD1-transfected group within 72 h. The data are expressed as the mean ± SEM. Statistical significance was determined by Student’s *t*-test. Asterisks indicate significant differences (*p < 0.05) between two groups.

**
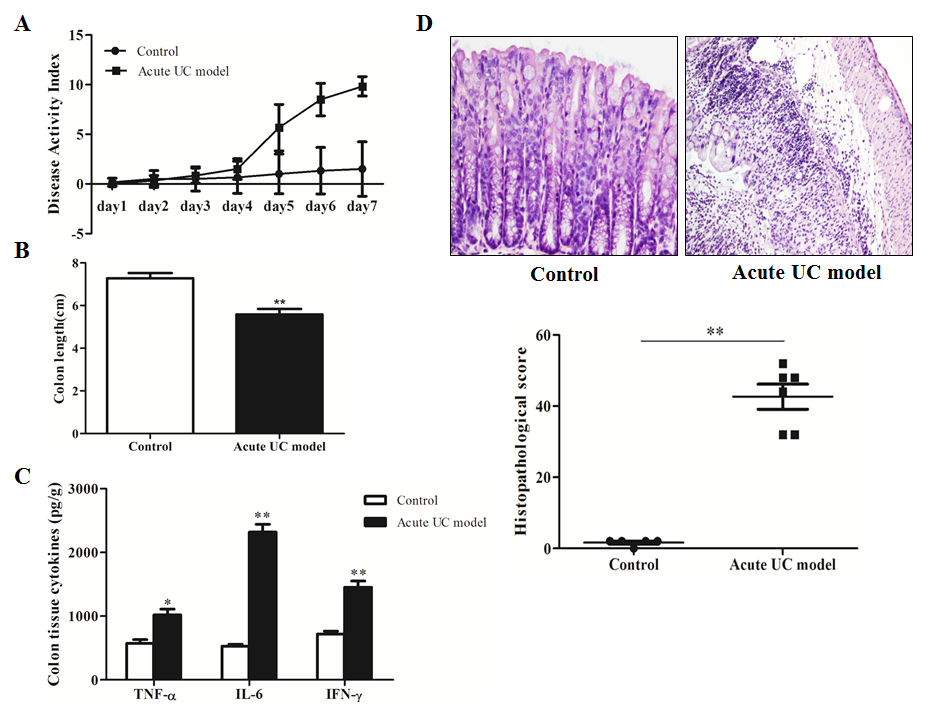
**

**Figure S5.** Establishment of DSS-induced colitis in mice. (A) Clinical assessment of DSS-induced UC-like colitis in mice. DAI was scored for each mouse from weight loss, faeces consistency and bleeding. The mean ± SD of at least five mice in each group are shown. (B) The length of the whole colon was measured. Control group (7.280 ± 0.2417, N=5) vs. DSS group (5.583 ± 0.2613, N=6), P=0.0011. The values are expressed as the mean ± SEM. (C) Evaluation of proinflammatory factors extracted from distal colon tissue. ELISA kits measured the secretion of TNF-α, IL-6 and IFN-γ by the colon. The results presented are representative of at least of three mice in each group. (D) Histological analysis of acute DSS-induced murine colitis model by H&E staining of colonic sections (Upper, magnification: 10*20). Scoring of murine experimental colitis was performed by detailed histological analysis, including inflammatory cell infiltration, extent, regeneration, crypt damage, and percent involvement of the colonic sections of each mouse. There was a significant increase in the histological scores after DSS exposure for 7 days (1.333 ±0.4216, N=6 vs. 42.67 ±3.528, N=6). The values are expressed as the mean ± SEM. Statistical P values were determined using a two-tailed Student’s *t*-test. **P<0.01.


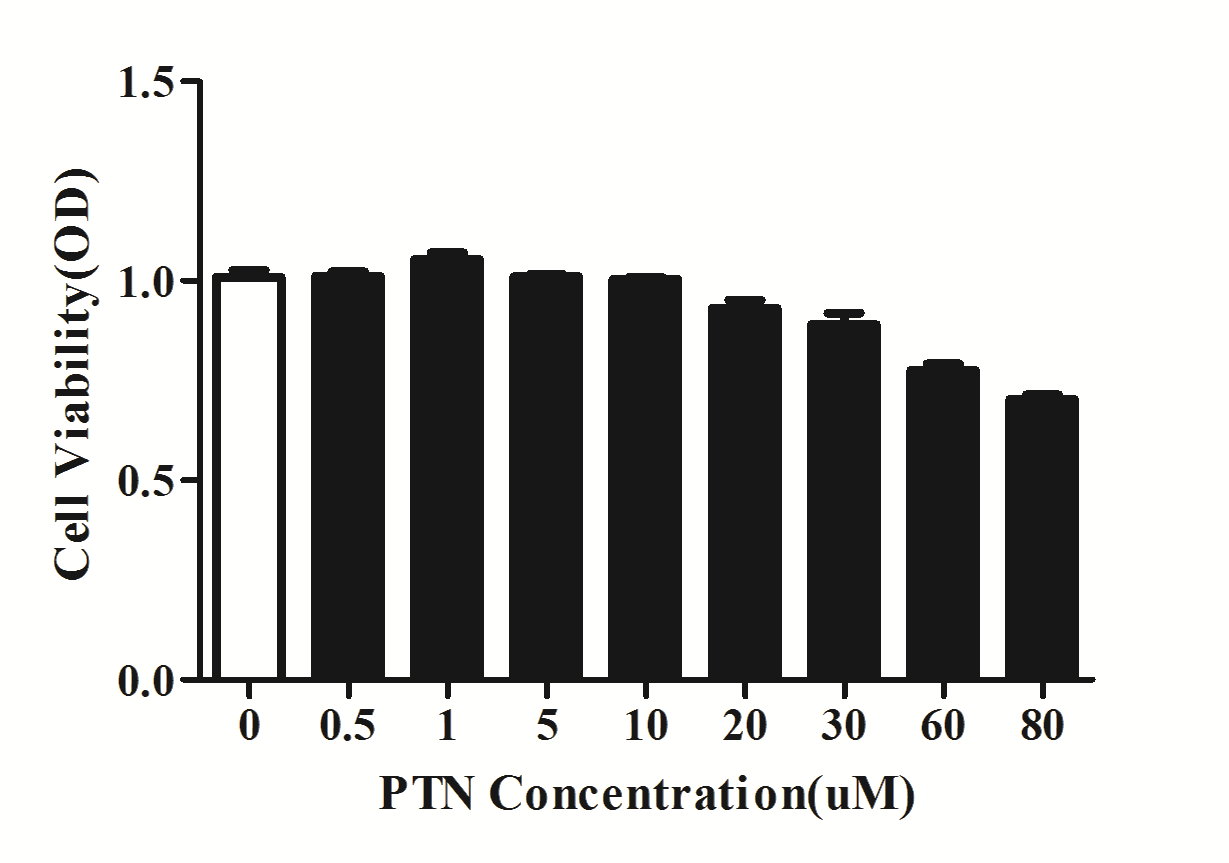


**Figure S6.** Effects of PTN on Caco-2 cell viability via CCK-8 assay. Caco-2 cells were incubated with different concentrations of PTN (0, 0.5, 1, 5, 10, 20, 30, 60, 80 µM) for 1 hour. At 24 h later, cell viability was determined by measuring the light absorbance at 450 nm with a microplate reader. The data shown are the mean ± SEM, and n=3 in each group.

**Supplementary Tables**

**Table S1. Distances between UC and different siRNA perturbations.**

| siRNA perturbation description | Distance value | siRNA perturbation description | Distance value |
| --- | --- | --- | --- |
| EZH2 | 0.868341908 | PTEN | 0.994893467 |
| UPF1 | 0.878459367 | NFE2L1 | 0.99686137 |
| FOXM1 | 0.8830626 | VIL2 | 0.997504894 |
| NUDT6 | 0.884844981 | NMNAT1 | 0.998303893 |
| BAHD1 | 0.890508081 | CXCR4 | 1.001097883 |
| BRCA1 | 0.891407694 | TFAP2C | 1.002634418 |
| EWSR1/FLI1 | 0.897273845 | SUZ12 | 1.004940815 |
| MMP14 | 0.899974733 | RBM21 | 1.00739654 |
| MYB | 0.90276804 | POU5F1 | 1.00767084 |
| PTK2 | 0.910692921 | AOF1 | 1.009320282 |
| PROX1 | 0.910804234 | SDHB | 1.00941293 |
| BMI1 | 0.911129752 | NRIP1 | 1.012587298 |
| FAS | 0.912065559 | IFITM1 | 1.013420897 |
| ARHGDIB | 0.925418621 | MYC | 1.013943319 |
| ERG | 0.929354883 | FOXF2 | 1.01706738 |
| SOX2 | 0.933656499 | PIR | 1.017135443 |
| ALDH1A1/ALDH1B1 | 0.934635329 | DSG2 | 1.01744275 |
| CREB1 | 0.935833371 | FTMT | 1.018112224 |
| AOF2 | 0.939384248 | E2F4 | 1.018921238 |
| YAP1 | 0.940130435 | CCND1 | 1.019543364 |
| YY2 | 0.940165718 | MAF | 1.023919417 |
| TARDBP | 0.941523105 | PDK2 | 1.030261552 |
| BIRC4 | 0.942736854 | FAP | 1.030616435 |
| SOX4 | 0.944014341 | PAX3 | 1.031188709 |
| PROM1 | 0.946888687 | SMAD2 | 1.034943547 |
| CTNNB1 | 0.949556795 | STAU1 | 1.035521739 |
| DNMT1 | 0.951748463 | KISS1R | 1.03821079 |
| SMAD3 | 0.951860915 | RBL2 | 1.038583201 |
| SNCA | 0.952601639 | WASF3 | 1.038859777 |
| CBFA2T3 | 0.954788755 | TP53 | 1.042501935 |
| QKI | 0.955205099 | ESR1 | 1.047104257 |
| BRAF | 0.958951059 | SRA1 | 1.048176645 |
| NME2 | 0.960077396 | SRF | 1.052368996 |
| TP73L | 0.961468017 | HDAC5 | 1.052954701 |
| POLRMT | 0.962323242 | EPAS1 | 1.053985887 |
| WTAP | 0.965204871 | EGR3 | 1.058866378 |
| YY1 | 0.966986342 | ETS1 | 1.060490326 |
| HOXC6 | 0.970587526 | HIF1A | 1.062743911 |
| PCSK9 | 0.97576804 | MYST1 | 1.066498065 |
| AR | 0.978225814 | CDR2 | 1.071099021 |
| RHOBTB2 | 0.978840883 | RB1 | 1.072946506 |
| CKS1B | 0.983101525 | NRAS | 1.07320214 |
| MITF | 0.986256772 | CIITA | 1.073802868 |
| PTHLH | 0.986589574 | IGF2BP1 | 1.078069201 |
| FSCN1 | 0.988584794 | CDX1 | 1.078575916 |
| DHX36 | 0.989030503 | PARK7 | 1.081597086 |
| PCGF2 | 0.989844981 | MCAM | 1.085606875 |
| CDH11 | 0.990228318 | PSIP1 | 1.090308218 |
| AHI1 | 0.990570453 | TFAP2A | 1.09405008 |
| PIP5K1A | 0.990811974 | WT1 | 1.09488163 |
| TOP1 | 0.992674937 | SCYL1 | 1.104386979 |
| CDK4 | 0.993578193 | CCNT1 | 1.119829046 |
| NR2F2 | 0.993816299 | BCL11A | 1.120596176 |

**Table S2. The expression profiles collected to characterize ulcerative colitis.**

| Instance No. | GEO Accession No. | | |
| --- | --- | --- | --- |
|  | Data Set | Health Sample | Patient Sample |
| Instance 1 | GSE3365 | GSM76115 | GSM76030 |
| Instance 2 | GSE3365 | GSM76116 | GSM76031 |
| Instance 3 | GSE3365 | GSM76117 | GSM76032 |
| Instance 4 | GSE3365 | GSM76118 | GSM76033 |
| Instance 5 | GSE3365 | GSM76119 | GSM76034 |
| Instance 6 | GSE3365 | GSM76120 | GSM76035 |
| Instance 7 | GSE3365 | GSM76121 | GSM76036 |
| Instance 8 | GSE3365 | GSM76122 | GSM76037 |
| Instance 9 | GSE3365 | GSM76123 | GSM76038 |
| Instance 10 | GSE3365 | GSM76124 | GSM76039 |
| Instance 11 | GSE3365 | GSM76125 | GSM76040 |
| Instance 12 | GSE3365 | GSM76126 | GSM76041 |
| Instance 13 | GSE3365 | GSM76127 | GSM76042 |
| Instance 14 | GSE3365 | GSM76128 | GSM76043 |
| Instance 15 | GSE3365 | GSM76129 | GSM76044 |
| Instance 16 | GSE3365 | GSM76130 | GSM76045 |
| Instance 17 | GSE3365 | GSM76131 | GSM76046 |
| Instance 18 | GSE3365 | GSM76132 | GSM76047 |
| Instance 19 | GSE3365 | GSM76133 | GSM76048 |
| Instance 20 | GSE3365 | GSM76134 | GSM76049 |
| Instance 21 | GSE3365 | GSM76135 | GSM76050 |
| Instance 22 | GSE3365 | GSM76136 | GSM76051 |
| Instance 23 | GSE3365 | GSM76137 | GSM76052 |
| Instance 24 | GSE3365 | GSM76138 | GSM76053 |
| Instance 25 | GSE3365 | GSM76139 | GSM76054 |
| Instance 26 | GSE3365 | GSM76140 | GSM76055 |

An instance contains a pair of samples collected from a patient and a healthy individual.

**Table S3. The expression profiles collected to characterize siRNA perturbations.**

| Gene Name | Accesion No. | Cell Type |
| --- | --- | --- |
| AHI1 | GSE14746 | Hut 78 cells |
| ALDH | GSE8045 | A549 lung cancer cell line |
| AR | GSE11428 | prostate cancer cells LNCaP and abl |
|  | GSE22483 | LNCaP |
| ARHGDIB | GSE8087 | MDA-MB-231 breast cancer cells |
| BAHD1 | GSE16097 | HEK293 cells |
| BCL11A | GSE13284 | erythroid progenitors |
| BMI1 | GSE21912 | RPMI-8226 human multiple myeloma cell line |
|  | GSE6015 | human embryonic fibroblast cells |
|  | GSE7578 | human medulloblastoma DAOY cell |
| BRAF | GSE13487 | melanoma A375 |
| BRCA1 | GSE22259 | HeLa cells |
|  | GSE4750 | MCF10A cells |
| CBFA2T3 | GSE20115 | acute lymphoblastic leukemia-derived Reh cells |
| CD133 | GSE24747 | CaCo-2 cell line |
| CD146 | GSE11951 | MDA-MB-231 |
| CDH11 | GSE14943 | MDA-MB-231 breast cancer cells |
| CDK4 | GSE8866 | IMR-32 |
| CDR2 | GSE20037 | Hela cells |
| CDX1 | GSE14380 | LS174T |
| CIITA | GSE25987 | KM-H2 cells |
| CKS1B | GSE3369 | Human Myeloma Cellline JJN3 |
| C-MYB | GSE13110 | human CD34+ Hematopoietic stem/progenitor cells |
|  | GSE21943 | CD14- myeloblasts |
| C-MYC | GSE5823 | BT-474, MCF-7, MDA-MB-231 and HeLa |
| CREB | GSE12056 | K562 myeloid leukemia cell line |
| CTNNB1 | GSE17385 | MM1.S cells |
| CXCR4 | GSE13763 | ovarian cancer cell line IGROV-1 |
| CYCLIN D1 | GSE8866 | IMR-32 |
| CYCLIN T1 | GSE10232 | Jurkat |
|  | GSE10233 | MM6 |
|  | GSE10234 | Jurkat |
|  | GSE10737 | Jurkat |
|  | GSE10738 | MM6 |
| DNMT1 | GSE18590 | epidermal progenitor cell |
| DSG2 | GSE21547 | human microvascular endothelial cells in healthy subjests |
| DZNEP | GSE18150 | Gliospheres |
| E2F4 | GSE19864 | Growing cells |
| EGR3 | GSE18913 | HUVEC |
| ERG | GSE14801 | HUVEC |
| ESR1 | GSE10890 | breast cancer cell line |
| ETS1 | GSE11710 | HaCaT |
| EWS/FLI | GSE14543 | Ewing's sarcoma cell lines (WE68, SK-N-MC, TC252, STA-ET-1, STA-ET-7.2) |
|  | GSE4560 | A673 Ewing's sarcoma cells |
|  | GSE7007 | Ewing cell lines; mesenchymal stem cells (MSC) |
| EZH2 | GSE12692 | A673 |
|  | GSE6015 | human embryonic fibroblast cells |
| EZRIN | GSE11279 | SW480 cells |
|  | GSE6233 | EC109, an esophageal squamous carcinoma cell line |
| FAK | GSE11581 | MCF-7 cell lines |
| FAP | GSE1773 | human melanoma LOX cell |
| FAS | GSE26594 | lung fibroblast cell line MRC5 |
| FASCIN | GSE11373 | esophageal squamous cell carcinoma (ESCC) |
| FGF-AS | GSE20070 | lung adenocarcinoma cell line Seg-1 |
| FOXF2 | GSE12444 | prostate stromal cells (PrSC) |
| FOXM1 | GSE2222 | BT-20 breast cancer cells |
| FTMT | GSE6817 | SK-Mel-28 |
| GPR54 | GSE25458 | Ishikawa |
| HDAC5 | GSE15499 | HUVEC |
| HIF-1alpha | GSE3188 | MCF7 |
|  | GSE7835 | U251 cells |
| HIF-2alpha | GSE3188 | MCF7 |
| HOXC6 | GSE9182 | LNCaP cells |
| IFITM1 | GSE20923 | HT-29 |
| IGF2BP1 | GSE21575 | HEK 293 cells |
| LSD1 | GSE13273 | neuroblastic tumors, SH-SY5Y cells |
| LSD2 | GSE22859 | HeLa Cell lines |
| MAF | GSE16356 | LEC cells |
| MITF | GSE16249 | NZM15 |
| MMP14 | GSE1774 | human fibrosarcoma cell line |
| MYST1 | GSE20193 | HEK293 cell line |
| NME2 | GSE18182 | A549 |
| NMNAT1 | GSE13458 | MCF-7 breast cancer cells |
| NR2F2 | GSE12846 | lymphatic endothelial cells |
| NRAS | GSE12445 | melanoma cell lines 224 and BL |
| PARK7 | GSE5519 | H157 cells |
| PAX3 | GSE16249 | NZM15 |
| PCGF2 | GSE7578 | human medulloblastoma DAOY cell |
| PCSK9 | GSE14434 | HeLa cells |
| PDK2 | GSE6014 | glioblastoma cells and lung carcinoma cells |
| PIPKIA | GSE9361 | HEK293 cells |
| PIR | GSE16798 | U937 cells |
|  | GSE17551 | melanoma cell lines |
| POLRMT | GSE2478 | Rho0-HeLa |
| POU5F1 | GSE12320 | GBS6 cells |
| PROX1 | GSE12846 | lymphatic endothelial cells |
| PSIP1 | GSE3485 | 293T derived cell line |
| PTEN | GSE7562 | A431 HCC827 SKBR3 |
| PTHRP | GSE4292 | MDA-MB-231 cells |
| QKI | GSE21574 | HEK 293 cells |
| RB | GSE19864 | Growing cells |
| RBL2 | GSE19864 | Growing cells |
| RHAU | GSE8192 | HeLa cell lines |
| RHOBTB2 | GSE8837 | NHBE |
| RIP140 | GSE7500 | human embryonal carcinoma cells (EC) |
| SCY1L1 | GSE23103 | HeLa |
| SDHB | GSE10289 | hepatocellular carcinoma cell line Hep3B cells |
| SMAD2 | GSE11710 | HaCaT |
| SMAD3 | GSE11710 | HaCaT |
| SNCA | GSE9807 | dopaminergic neuroblastoma cells |
| SOX2 | GSE20689 | SW620 |
| SOX4 | GSE11914 | LNCaP |
|  | GSE4225 | The ACC3 cell line |
| SRA | GSE20081 | Hela cells, MCF-7 |
| SRF | GSE22606 | LNCaP cells |
| STAU1 | GSE6679 | HeLa cells |
| SUZ12 | GSE6015 | human embryonic fibroblast cells |
| TARDBP | GSE18632 | HEK293E cells |
| TCF11 | GSE22853 | Ea.hy926 cells |
| TFAP2A | GSE11710 | HaCaT |
|  | GSE8640 | MCF7 cells |
| TFAP2C | GSE15481 | MCF-7 |
|  | GSE8640 | MCF7 cells |
| TOP1 | GSE19445 | LNCaP cells |
|  | GSE7161 | HCT116,MCF-7 |
| TP53 | GSE14491 | MDA MDA-MB-231with mutant p53 |
|  | GSE19670 | SKBR3 |
| TP63 | GSE5993 | ME180 cells |
| TUT1 | GSE9361 | HEK293 cells |
| UPF1 | GSE7009 | HeLa cells |
| WASF3 | GSE26462 | MDA-MB-231 cells |
| WT1 | GSE12886 | human CD34+ hematopoietic stem/progenitor cells  human hepatocellular carcinoma (HCC) cell lines |
| WTAP | GSE2327 | HUVEC |
| XIAP | GSE11618 | a parent HCT116 human colon cancer cell line |
| YAP | GSE7700 | normal breast luminal cell and breast cancer cell lines(MDA-MB-231) |
| YY1 | GSE14964 | HeLa cells |
| YY2 | GSE14964 | HeLa cells |

**Table S4. Primers used in the present study.**

| Primer | Sequence(5'--- 3') |
| --- | --- |
| human TNF-α Forward | GAGGCCAAGCCCTGGTATG |
| human TNF-α Reverse | CGGGCCGATTGATCTCAGC |
| human IL-6 Forward | ACTCACCTCTTCAGAACGAATTG |
| human IL-6 Reverse | CCATCTTTGGAAGGTTCAGGTTG |
| human IFN-γ Forward | TCGGTAACTGACTTGAATGTCCA |
| human IFN-γ Reverse | TCGCTTCCCTGTTTTAGCTGC |
| human IL-8 Forward | ACTGAGAGTGATTGAGAGTGGAC |
| human IL-8 Reverse | AACCCTCTGCACCCAGTTTTC |
| human ICAM-1 Forward | ATGCCCAGACATCTGTGTCC |
| human ICAM-1 Reverse | GGGGTCTCTATGCCCAACAA |
| human VCAM-1 Forward | GGGAAGATGGTCGTGATCCTT |
| human VCAM-1 Reverse | TCTGGGGTGGTCTCGATTTTA |
| human CCL3 Forward | AGTTCTCTGCATCACTTGCTG |
| human CCL3 Reverse | CGGCTTCGCTTGGTTAGGAA |
| human CCL4 Forward | CTGTGCTGATCCCAGTGAATC |
| human CCL4 Reverse | TCAGTTCAGTTCCAGGTCATACA |
| human TNFR1 Forward | TCACCGCTTCAGAAAACCACC |
| human TNFR1 Reverse | GGTCCACTGTGCAAGAAGAGA |
| human BAHD1 Forward | TAGCGAGGACACTGGAGTGAA |
| human BAHD1 Reverse | CCTTCTGCTGCAAAAGGCATT |
| human IL-1β Forward | AGCTACGAATCTCCGACCAC |
| human IL-1β Reverse | CGTTATCCCATGTGTCGAAGAA |
| human IFN-β Forward | ATGACCAACAAGTGTCTCCTCC |
| human IFN-β Reverse | GGAATCCAAGCAAGTTGTAGCTC |
| human CCL5 Forward | CCAGCAGTCGTCTTTGTCAC |
| human CCL5 Reverse | CTCTGGGTTGGCACACACTT |
| human CX3CL1 Forward | ACCACGGTGTGACGAAATG |
| human CX3CL1 Reverse | TGTTGATAGTGGATGAGCAAAGC |
| human CXCL10 Forward | GTGGCATTCAAGGAGTACCTC |
| human CXCL10 Reverse | TGATGGCCTTCGATTCTGGATT |
| human CXCL3 Forward | CGCCCAAACCGAAGTCATAG |
| human CXCL3 Reverse | GCTCCCCTTGTTCAGTATCTTTT |
| human CXCL5 Forward | AGCTGCGTTGCGTTTGTTTAC |
| human CXCL5 Reverse | TGGCGAACACTTGCAGATTAC |
| human iNOS Forward | TTCAGTATCACAACCTCAGCAAG |
| human iNOS Reverse | TGGACCTGCAAGTTAAAATCCC |
| human eNOS Forward | TGATGGCGAAGCGAGTGAAG |
| human eNOS Reverse | ACTCATCCATACACAGGACCC |
| human GAPDH Forward | TCAACGACCACTTTGTCAAGCTCA |
| human GAPDH Reverse | GCTGGTGGTCCAGGGGTCTTACT |
| mouse EZH2 Forward | AGCACAAGTCATCCCGTTAAAG |
| mouse EZH2 Reverse | AATTCTGTTGTAAGGGCGACC |
| mouse UPF1 Forward | GTGGCAGCCCCTAATCCAG |
| mouse UPF1 Reverse | GATCTGCTGTGCCGTGATCT |
| mouse FOXM1 Forward | CAGAATGCCCCGAGTGAAACA |
| mouse FOXM1 Reverse | GTGGGGTGGTTGATAATCTTGAT |
| mouse NUDT6 Forward | GCTCAGAAGGACGGATAGCTG |
| mouse NUDT6 Reverse | CAGTGTTGAGGAATGGGGTTTT |
| mouse BAHD1 Forward | AGCTACCTCTTCGACCTTCTC |
| mouse BAHD1 Reverse | CTTGCCGTTGACCTTTGGC |
| mouse beta-ACTIN Forward | GAAGATCAAGATCATTGCTCCT |
| mouse beta-ACTIN Reverser | TGGAAGGTGGACAGTGAG |

**Table S5. Histological grading of experimental colitis mouse model.**

| Feature graded | Grade | Description |
| --- | --- | --- |
| Inflammation | None | 0 |
|  | Slight | 1 |
|  | Moderate | 2 |
|  | Severe | 3 |
| Extent | None | 0 |
|  | Mucosa | 1 |
|  | Mucosa and submucosa | 2 |
|  | Transmural | 3 |
| Crypt damage | None | 0 |
|  | Basal 1/3 damaged | 1 |
|  | Basal 2/3 damaged | 2 |
|  | Only surface epithelium intact | 3 |
|  | Entire crypt and epithelium lost | 4 |
| Regeneration | Complete regeneration or normal tissue | 0 |
|  | Almost complete regeneration | 1 |
|  | Regeneration with crypt depletion | 2 |
|  | Surface epithelium not intact | 3 |
|  | No tissue repair | 4 |
| Percent involvement（%） | 1—25% | 1 |
|  | 26—50% | 2 |
|  | 51—75% | 3 |
|  | 76—100% | 4 |

**Disease Activity Index (DAI)** ^5^

The DAI was scored as follows: (1) weight loss (no change=0; 1–5%=1; 5–10%=2; 10–15%=3; >15%=4); (2) faeces (normal=0; pasty, semiformed=1; liquid, loose=3); (3) blood (no blood=0; occult blood (+)=1; occult blood (++)=2; visible blood in rectum (+++)=3; visible blood (++++)=4;).

**Histological Scoring of the Experimental Colitis Model**

Distal colons were excised from DSS-treated and control mice. Histological signs of inflammation were evaluated as a combined score of inflammatory cell infiltration (0-3), extent (0-3), regeneration (0-4), crypt damage (0-4), and percent involvement (0-4) as indicated in Supplementary Table S3 and as previously described^6^.

**REFERENCES**

1. Frey, B. J. & Dueck, D. Clustering by passing messages between data points. *Science*. **315**, 972-976 (2007).

2. Iorio, F. et al. Discovery of drug mode of action and drug repositioning from transcriptional responses. *P. Natl. Acad. Sci. USA*. **107**, 14621-14626 (2010).

3. Li, F. et al. GeneExpressionSignature: an R package for discovering functional connections using gene expression signatures. *Omics*. **17**, 116-118 (2013).

4. Ni, M. et al. ExpTreeDB: Web-based query and visualization of manually annotated gene expression profiling experiments of human and mouse from GEO. *Bioinformatics.* **30**, 3379-3386 (2014).

5. Cooper, H. S., Murthy, S. N., Shah, R. S. & Sedergran, D. J. Clinicopathologic study of dextran sulfate sodium experimental murine colitis. *Lab. Invest*. **69**, 238-249 (1993).

6. Dieleman, L. A. et al. Chronic experimental colitis induced by dextran sulphate sodium (DSS) is characterized by Th1 and Th2 cytokines. *Clin. Exp. Immunol*. **114**, 385-391 (1998).
